# Structural centrosome aberrations sensitize polarized epithelia to basal cell extrusion

**DOI:** 10.1098/rsob.180044

**Published:** 2018-06-13

**Authors:** Olivier Ganier, Dominik Schnerch, Erich A. Nigg

**Affiliations:** Biozentrum, University of Basel, Klingelbergstrasse 50/70, 4056 Basel, Switzerland

**Keywords:** structural centrosome aberrations, basal cell extrusion, NLP, CEP131, metastasis

## Abstract

Centrosome aberrations disrupt tissue architecture and may confer invasive properties to cancer cells. Here we show that structural centrosome aberrations, induced by overexpression of either Ninein-like protein (NLP) or CEP131/AZI1, sensitize polarized mammalian epithelia to basal cell extrusion. While unperturbed epithelia typically dispose of damaged cells through apical dissemination into luminal cavities, certain oncogenic mutations cause a switch in directionality towards basal cell extrusion, raising the potential for metastatic cell dissemination. Here we report that NLP-induced centrosome aberrations trigger the preferential extrusion of damaged cells towards the basal surface of epithelial monolayers. This switch in directionality from apical to basal dissemination coincides with a profound reorganization of the microtubule cytoskeleton, which in turn prevents the contractile ring repositioning that is required to support extrusion towards the apical surface. While the basal extrusion of cells harbouring NLP-induced centrosome aberrations requires exogenously induced cell damage, structural centrosome aberrations induced by excess CEP131 trigger the spontaneous dissemination of dying cells towards the basal surface from MDCK cysts. Thus, similar to oncogenic mutations, structural centrosome aberrations can favour basal extrusion of damaged cells from polarized epithelia. Assuming that additional mutations may promote cell survival, this process could sensitize epithelia to disseminate potentially metastatic cells.

## 1. Introduction

Centrosomes are the major microtubule (MT) organizing centres in animal cells and typically comprise two centrioles embedded in a pericentriolar matrix (PCM) [1–5]. While centrosome numbers are strictly controlled during proliferation of healthy cells [6,7], cancer cells frequently show centrosome amplification or structural centrosome aberrations [8–11]. Furthermore, experimental manipulation of centrosome numbers can trigger tumorigenesis in flies [12] as well as mice [13–15]. This raises the question of whether centrosome aberrations contribute to cancer development or progression, and, if so, through what mechanisms. Evidence suggests that centrosome aberrations may contribute to aneuploidy as well as disruption of tissue architecture [10,11,16], two hallmarks of human cancers [17].

 In most animal cells, centrosomes play important roles in the assembly and positioning of mitotic spindles. Accordingly, much of the early work in the field focused on the impact of centrosome aberrations on the fidelity of chromosome segregation. It is now widely accepted that numerical centrosome aberrations increase the frequency of chromosome mis-segregation, thereby causing aneuploidy and chromosomal instability (e.g. [15,18,19]. More recent studies have also begun to explore the impact of centrosome aberrations on tissue architecture and, potentially, metastatic invasion [16,20–23]. Most of these studies made use of three-dimensional (3D) culture models based on acini derived from human breast epithelial MCF10A cells or cysts derived from canine kidney epithelial MDCK cells. One favourite approach for inducing numerical centrosome aberrations consists in the overexpression of Polo-like kinase 4 (PLK4), the master regulator of centriole biogenesis [24,25]. Similarly, structural centrosome aberrations closely resembling those seen in tumours can be triggered by overexpression of Ninein-like protein (NLP), a PCM component implicated in MT anchoring during interphase of the cell cycle [21,23,26]. NLP is frequently overexpressed in human cancers, and its overexpression in transgenic mice was reported to cause tumorigenesis [27].

A first important mechanism through which centrosome aberrations may confer invasive properties relates to the formation of invadopodia. This phenotype was originally discovered in a study focusing on the consequences of PLK4-induced centrosome amplification [20]. Subsequent analyses showed that invadopodia formation can also be triggered by structural centrosome aberrations, suggesting that it may constitute a more widespread response to centrosome aberrations [23]. A second, mechanistically distinct mechanism of potential relevance to metastatic cell dissemination was discovered in a study focusing on the impact of NLP-induced structural centrosome aberrations. Specifically, NLP-induced centrosome aberrations were shown to stimulate the dissemination (budding) of mitotic cells from 3D acini and cysts through a non-cell-autonomous process [23]. This phenotype was attributed to an impairment of E-cadherin-mediated cell–cell interactions, combined with an increased stiffness of cells harbouring NLP-induced centrosome aberrations. Most importantly, dividing cells were found to be extruded from epithelia, regardless of whether or not they themselves harbour centrosome aberrations. Thus, the proposed non-cell-autonomous mechanism holds the potential to answer a long-standing conundrum in the field and explain how centrosome aberrations may confer a selective advantage to tumour cells, even though they are *a priori* expected to impair cell viability [16,23].

In this study, we have explored a possible connection between centrosome aberrations and ‘basal cell extrusion', another fundamental mechanism implicated in the dissemination of metastatic cells [28,29]. To the best of our knowledge, a possible connection between centrosome aberrations and basal cell extrusion has not previously been explored. Cell extrusion is an important process through which epithelia respond to overcrowding or cell damage [29]. In fact, the removal of aberrant cells, followed by gap closure by neighbouring healthy cells, is critical to preserve the integrity of epithelial layers [28,29]. In normally polarized mammalian epithelia, aberrant or dying cells are typically extruded at the apical side, resulting in their efficient elimination via the lumen of the cavity [28]. By contrast, a conspicuous change in the directionality of extrusion has been observed in cancer [28,30]. This alteration of directionality in favour of basal extrusion interferes with the elimination of aberrant or dying cells into the glandular lumen and, instead, favours the accumulation of extruded cells underneath the epithelial sheet [28,30]. It has therefore been argued that basally extruded cells may harbour or acquire oncogenic alterations, which may then allow them to survive and persist in a juxta-epithelial position. Having escaped the context of an intact epithelium, basally extruded cells may accumulate additional genetic changes that enable them to travel through the extracellular matrix, potentially seeding metastatic disease [28–31]. In support of this hypothesis, mutant K-Ras provides an enhanced survival signal and promotes invasive behaviour of extruded cells [32]. In addition, highly metastatic cancers, notably pancreatic cancers harbouring a mutant K-Ras protein, exhibit a strong bias in favour of basal extrusion [33]. Similarly, mutant versions of the tumour suppressor gene product adenomatous polyposis coli (APC) were also shown to favour a reversal in the directionality of cell extrusion, and this was attributed to APC's role in controlling the disposition of MTs and cortical actin within the extruded cell [28,34]. Collectively, these findings support the hypothesis that an evolutionarily conserved mechanism for the removal of damaged cells from otherwise healthy epithelia can be subverted by oncogenically mutated cells to favour metastatic cell dissemination [28].

The observation that basal cell extrusion requires the MT cytoskeleton [34,35] prompted us to ask whether centrosome aberrations might exert an influence on the directionality of cell extrusion from epithelial layers. Following up on earlier work [21,23], we focused primarily on structural centrosome aberrations induced by overexpression of NLP. In addition, we examined the consequences of centrosome aberrations induced by excess CEP131 (also known as AZI1), a centrosomal protein that is also frequently overexpressed in cancer [36,37]. Although the structural centrosome aberrations induced by excess NLP or CEP131 display distinct properties, we found that both types of aberrations influence the directionality of extrusion of damaged cells from epithelia. This leads us to conclude that centrosome aberrations, much like previously described oncogenic mutations, can confer a bias towards basal cell extrusion. This unexpected impact of aberrant centrosomes on the directionality of cell extrusion from epithelial layers offers a new perspective on the possible contributions of centrosome aberrations to metastasis.

## 2. Results

### 2.1. Directionality of cell extrusion from three-dimensional MDCK cysts

**Figure 1.**
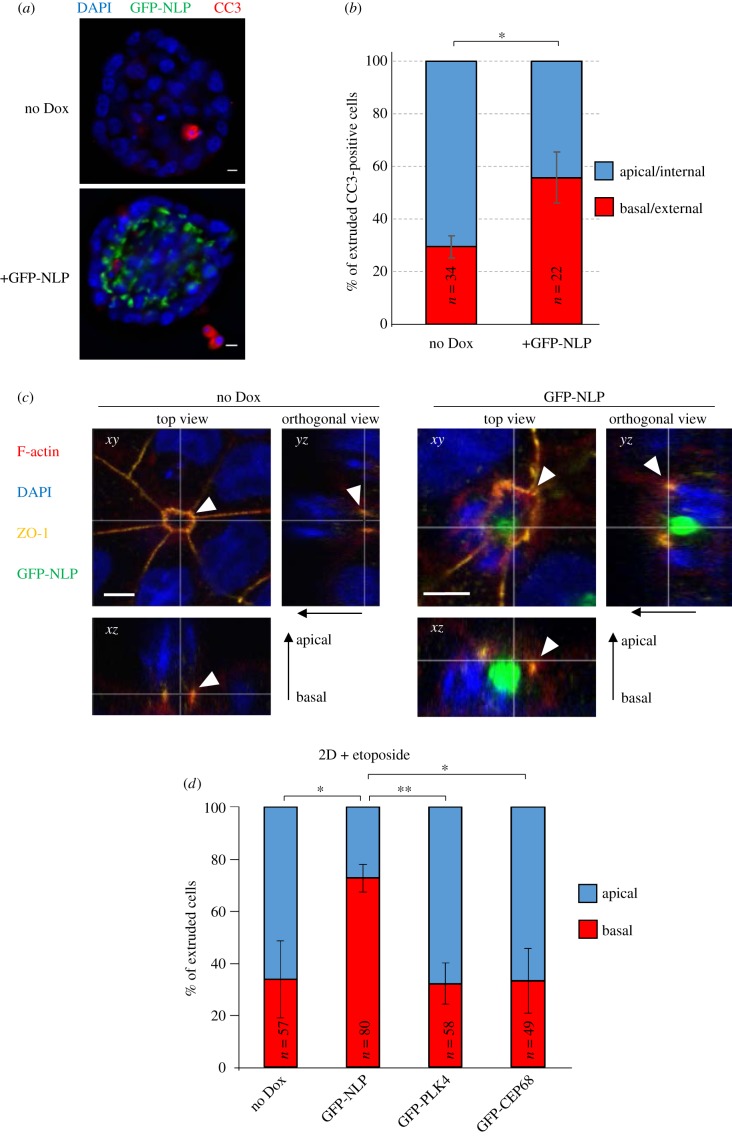
NLP-induced centrosome aberrations interfere with the directionality of extrusion. (*a*) Representative images show MDCK-derived cysts expressing GFP-NLP (lower panel) or not (upper panel) after staining for cleaved caspase 3 (CC3; red). GFP-NLP is shown in green and DNA in blue (DAPI staining). Scale bars = 10 µm. (*b*) Histogram indicates the mean percentages of apoptotic, CC3-positive cells extruding basally (red) versus apically (blue) from MDCK cysts induced (GFP-NLP) or not (no Dox) to express GFP-NLP, as illustrated in (a). CC3-positive cells were classified as apical/internal or basal/external, depending on their position relative to the outermost layer of cells delineating the cyst. *n* refers to the numbers of CC3-positive cells detected in 155 control cysts (no Dox) and 111 GFP-NLP expressing cysts (compiled from three independent experiments); error bars indicate standard deviation and *p*-values were derived from unpaired, two-tailed Student's *t*-test. (*) indicates *p* < 0.05. We note that the data shown in figure 1*b* are also used in figure 7*b,c* (different display, same series of experiments). (*c*) Representative confocal microscopy images show extrusion of damaged cells from 2D MDCK monolayer cultures, induced (GFP-NLP) or not (no Dox) for 24 h to express GFP-NLP, and then treated with etoposide. Cells were fixed, permeabilized, stained as indicated and examined by confocal fluorescence microscopy. GFP-NLP is shown in green and DNA in blue (DAPI staining). F-actin is pseudo-coloured in red and ZO-1 in yellow. Main panels show top views (*xy* sections). Corresponding orthogonal sections derived from 3D reconstructions of *z*-stacks are shown below (*xz*) and to the right (*yz*). The thin white lines illustrate the positions of the optical sections and white arrowheads point to the actomyosin rings; epithelial polarity is indicated by arrows (apical/basal). Note that F-actin and ZO-1 co-localize during actomyosin ring closure and extrusion of the damaged cell. Scale bars = 5 µm. (*d*) Histogram indicates the mean percentages of cells extruding basally (red bars) versus apically (blue bars) from 2D cultures of MDCK cells. MDCK cells were induced or not (no Dox) to express the indicated transgenes (GFP-NLP, GFP-PLK4 or GFP-CEP68) for 24 h and treated with etoposide. *n* refers to the numbers of extruded cells (compiled from three independent experiments); error bars indicate standard deviation and *p*-values were derived from unpaired, two-tailed Student's *t*-test. (*) indicates *p* < 0.05 and (**) indicates *p* < 0.01.

While exploring the consequences of centrosome aberrations on the 3D architecture of MCF10A spheroids and MDCK cysts, we had noticed occasional occurrence of dissemination of dying cells [23]. In consideration of the potential importance of basal cell extrusion for metastasis [28,29], this led us to ask whether NLP-induced centrosome aberrations might affect the directionality of extrusion of dying cells. As determined by staining of MDCK cells for CC3, a marker of apoptosis [38–40], overexpression of NLP did not *per se* affect the frequency of cell death (electronic supplementary material, figure S1a). However, while in control MDCK cysts a majority of CC3-positive cells were observed in the interior of the cysts, consistent with apical extrusion, the expression of GFP-NLP induced a significant bias in favour of basal cell extrusion towards the matrix, resulting in CC3-positive cells immediately adjacent to the cysts (figure 1*a,b*). We recognize that analyses of fixed samples cannot definitively distinguish extrusion of dying cells from death occurring *in situ*, but we are confident that the majority of CC3-positive cells counted in the fixed cysts reflect extrusion events (see results of live-cell imaging experiments). To overcome limitations imposed by the scarcity of spontaneously occurring cell death and explore this phenomenon of directionality reversal more systematically, we next adopted an experimental protocol that increases the number of damaged cells available for analysis. This protocol involves treatment of epithelia with the DNA-damaging drug etoposide and is commonly used in studies on basal cell extrusion [33,34]. In line with earlier results [34], etoposide treatment reproducibly caused occasional cell death, as visualized by CC3 staining, in otherwise healthy epithelia (electronic supplementary material, figure S1b).

### 2.2. Directionality of cell extrusion in polarized two-dimensional MDCK cultures

The mechanisms controlling the directionality of cell extrusion from polarized epithelia have been studied extensively in 2D cultures of MDCK cells [33–35]. Thus, we next examined the influence of NLP-induced centrosome aberrations on the directionality of cell extrusion from polarized epithelial MDCK monolayers. The induction of NLP transgene expression had no detectable effect on cell viability and, likewise, the level of programmed cell death triggered by detachment of MDCK cells from the substratum (anoikis) was unaffected by NLP expression (electronic supplementary material, figure S1a). However, when MDCK cells were treated with etoposide, we observed the formation of characteristic rosette structures (figure 1c; electronic supplementary material, figure S2). As shown previously [33,34], these rosettes arise when dying cells are pushed out of the epithelial layer by surrounding normal cells (see also electronic supplementary material, figure S1b). To monitor the placement of actomyosin ring formation and sealing of tight junctions relative to the position of the nucleus within the cell about to be extruded, we stained MDCK monolayers for F-actin and Zona occludens-1 (ZO-1) protein [33] and nuclear DNA with DAPI (figure 1c). In control MDCK cells (no Dox), actomyosin/ZO-1 ring formation occurred close to the substratum, resulting in ring closure underneath the nucleus and extrusion of the cell towards the apical side of the monolayer (figure 1c; electronic supplementary material, figures S1 and S3). In striking contrast, in cells expressing NLP-induced structural centrosome aberrations, formation of the actomyosin/ZO-1 rings frequently occurred above the nucleus of the cell, leading to basal extrusion (figure 1c, right panels; see also electronic supplementary material, figure S1–S3). Quantification of these data showed that 73% of cells expressing GPF-NLP underwent basal extrusion, as compared with 34% of cells in the control cultures (figure 1*d*). Interestingly, this reversal in the directionality of cell extrusion was not seen in response to overexpression of either PLK4, the master regulator of centriole duplication [24,25], or CEP68, a protein implicated in centrosome cohesion [41] (figure 1*d*). While overexpression of NLP causes structural centrosome aberrations, PLK4 overexpression triggers numerical centrosome aberrations and excess of CEP68 causes no detectable aberrations [21,23]. This suggests that the NLP-induced predisposition for basal cell extrusion probably represents a specific response to structural centrosome aberrations.

**Figure 2.**
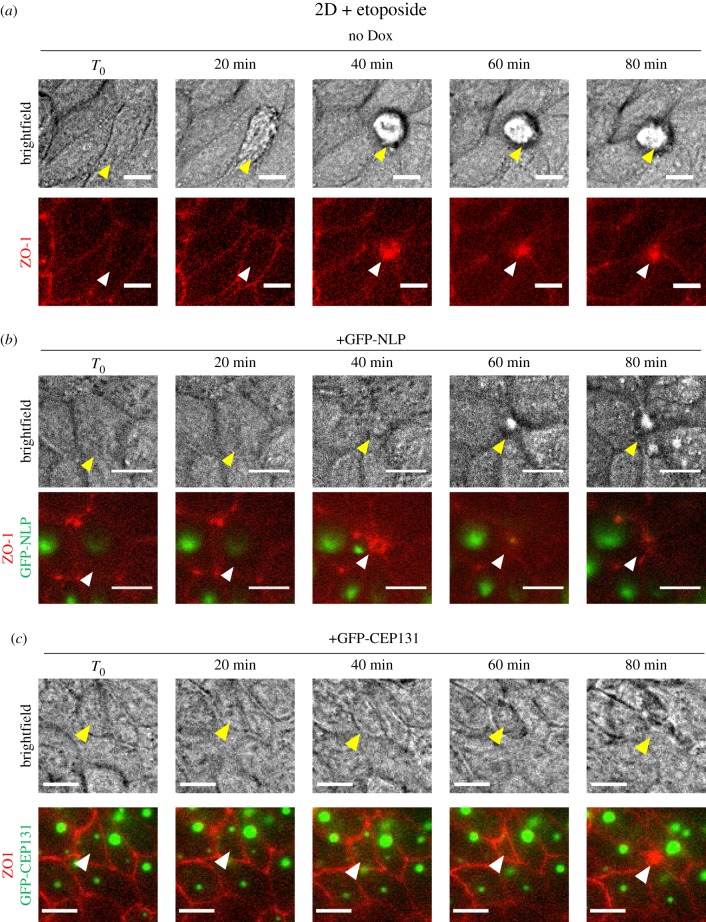
Monitoring of apical and basal extrusion by time-lapse microscopy. Still series from time-lapse experiments performed on 2D MDCK monolayer cultures (expressing mCardinal-ZO-1 and the indicated transgenes). Cells were induced to express transgenes for 48 h and then treated with etoposide. (*a*) Control MDCK cells (no Dox); (*b*) MDCK cells expressing GFP-NLP; (*c*) MDCK cells expressing GFP-CEP131. Time lapse was started immediately after etoposide addition and images were acquired every 20 min. *T*_0_ was arbitrarily defined as the first frame prior to clear evidence for the onset of cell extrusion. Upper panels show brightfield images (top views), illustrating the directionality of cell extrusion. Note that extruded cells appear above the monolayer only in case of apical extrusion (*a*), but not in case of basal extrusion (*b*) and (*c*); extruding cells are marked by yellow arrowheads. Lower panels show corresponding fluorescence images (GFP-NLP or GFP-CEP131 in green and mCardinal-ZO-1 in red), illustrating closure of ZO-1 junctions (white arrowheads). Scale bars = 10 µm.

To corroborate the above conclusion, time-lapse microscopy was performed on MDCK cells stably expressing mCardinal-ZO-1. This allowed us to visualize cell extrusion and closure of tight junctions in etoposide-treated cultures (figure 2; electronic supplementary material, movies S1 and S2). Upon treatment of control MDCK cells with etoposide (no Dox), damaged cells were seen by brightfield microscopy to undergo apical extrusion, resulting in their progressive appearance above the plane of the monolayer (figure 2*a*, upper panels; electronic supplementary material, figure S1). Concomitantly, contraction of ZO-1 rings could clearly be observed (figure 2*a*, lower panels), resulting in typical rosette formation [33,35]. By contrast, etoposide treatment of MDCK cells harbouring NLP-induced centrosome aberrations resulted in basal extrusion of damaged cells, as revealed by the apparent disappearance of the extruded cell when viewing the epithelium from the apical surface (figure 2*b*, upper panels; electronic supplementary material, movie S2). Again, cell extrusion was accompanied by closure of the ZO-1 ring (figure 2*b*, lower panels).

**Figure 3.**
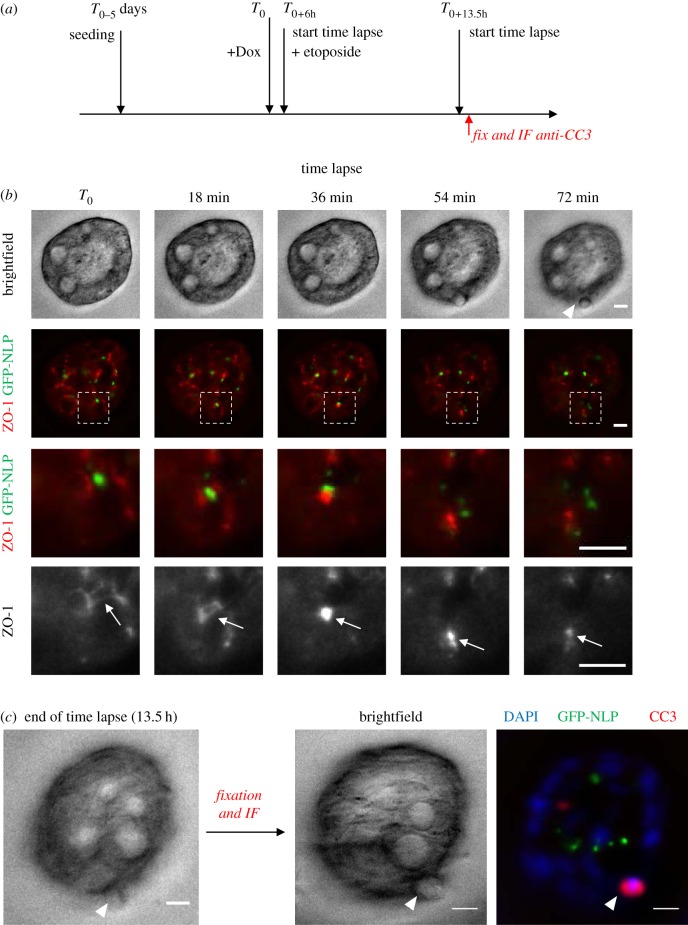
GFP-NLP overexpression triggers basal extrusion of dying cells from MDCK cysts. (*a*) Schematic description of experiments. After induction of GFP-NLP in MDCK cysts and monitoring of etoposide-induced basal cell extrusion events by time-lapse microscopy, cysts were fixed and subjected to analysis by fluorescence microscopy. In total, six events of basal cell extrusion from distinct cysts were recorded. (*b*) Still series from a representative time-lapse experiment showing basal extrusion of a cell from a MDCK cyst constitutively expressing mCardinal-ZO-1 and induced to express GFP-NLP. Shown are brightfield images (first row) and fluorescence images (second to fourth row) illustrating either merged GFP-NLP (green) and mCardinal-ZO-1 (red) signals (second and third rows) or mCardinal-ZO-1 alone (fourth row). Dashed squares (second row) indicate the regions chosen for enlargements, shown below each image (third and fourth row). White arrowhead points to extruded cell and white arrows to ZO-1 ring closure. Images were acquired every 18 min and time stamps are indicated. Scale bars= 10 µm. (*c*) At the end of time-lapse imaging, cysts were fixed and processed for immunofluorescence microscopy (IF). Corresponding cysts were identified through use of gridded coverslips. IF was performed using anti-CC3 antibodies to detect apoptotic cells. The two brightfield images show the same cyst, once as recorded by time-lapse (last frame; left panel) and once after fixation (central panel); arrowheads point to the extruded cell. The right panel shows merged fluorescence images of the same cyst, illustrating DNA (blue), GFP-NLP (green) and CC3 (red). Scale bars= 10 µm.

To extend this analysis to 3D epithelial structures and rigorously demonstrate that basal extrusion triggered by NLP-induced centrosome aberrations concerns damaged cells, we combined time-lapse microscopy on etoposide-treated MDCK cysts with fixation of the same cysts, followed by staining for CC3 (figure 3*a*). While bright-field microscopy allowed us to monitor basal cell extrusion into the surrounding matrix, GFP-NLP and mCardinal-ZO-1 fluorescence signals allowed us to visualize centrosome aberrations and junction ring closure, respectively (figure 3*b*; electronic supplementary material, movie S3). Finally, immunofluorescence staining of the very same cysts for CC3 demonstrated that the extruded cells were indeed undergoing apoptosis (figure 3*c*). These results support the conclusion that NLP-induced structural centrosome aberrations sensitize epithelial cells to undergo basal extrusion in response to etoposide-induced damage.

### 2.3. NLP-induced basal extrusion is controlled by SP1-S1PR2 signalling

**Figure 4.**
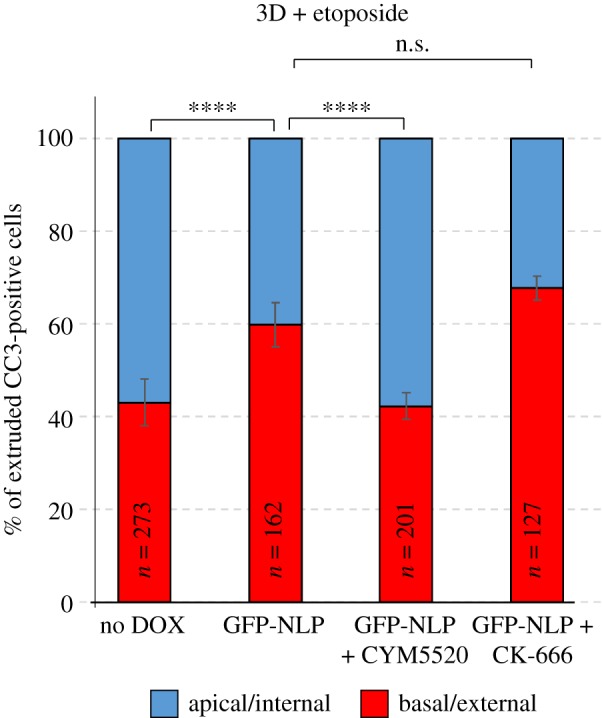
NLP-induced basal cell extrusion involves S1P-S1PR2 signalling. Histogram indicates the mean percentages of cells extruded basally (red bars) versus apically (blue bars) from MDCK cysts, as defined in figure 1. MDCK cysts were induced for 48 h to express GFP-NLP or not (no Dox) and then treated with etoposide for 16 h. Where indicated, they were simultaneously treated with CYM-5520, an agonist of S1PR2, or CK-666, an inhibitor of Arp2/3 for control. *n* refers to the numbers of CC3-positive cells (compiled from three independent experiments); error bars indicate standard deviation and *p*-values were derived from unpaired, two-tailed Student's *t*-test. n.s. indicates not significant; (****) indicates *p* < 0.0001.

The above data raised the question of whether the basal extrusion events triggered by NLP-induced centrosome aberrations might be mechanistically related to those observed in MDCK cysts expressing mutant K-Ras [33]. In previous work, mutant K-Ras was shown to cause reversal of extrusion directionality through downregulation of the lipid sphingosine 1-phosphate (S1P) and its receptor sphingosine 1-phosphate receptor 2 (S1PR2), both of which are required for apical extrusion [33]. To determine whether a similar pathway operates when basal extrusion is triggered by structural centrosome aberrations, we asked whether modulation of S1P signalling would interfere with the directionality of extrusion of cells harbouring excess NLP. To this end, MDCK cysts were induced to overexpress NLP and incubated with or without the S1PR2-agonist CYM-5520 [31,42] or the Arp2/3 inhibitor CK-666 for control [43]. MDCK cysts were then challenged with etoposide and the directionality of cell extrusion determined by microscopy. Compared with control cysts (no Dox), the expression of GFP-NLP caused a significant bias in favour of basal extrusion over apical extrusion (figure 4), consistent with data obtained for 2D cultures (figure 1*d*). Remarkably, however, exposure of these GFP-NLP expressing cysts to CYM-5520 resulted in a marked reduction in the frequency of basal extrusions and a corresponding increase in the frequency of apical extrusions, essentially restoring the balance of directionalities observed in control cysts (figure 4). This indicates that the directionality reversal triggered by structural centrosome aberrations could be suppressed by restoration of S1P-S1PR2 signalling. Treatment of GFP-NLP-expressing cysts with CK-666 did not detectably affect the directionality of extrusion, attesting to the specificity of the effect caused by CYM-5520 (figure 4). These data indicate that NLP-induced structural centrosome aberrations cause a reversal of extrusion directionality through modulation of the S1P-S1PR2 signalling pathway, highly reminiscent of the mechanism activated in response to mutant K-Ras protein [33].

### 2.4. Basal extrusion correlates with apical localization of MTs and centrosome

**Figure 5.**
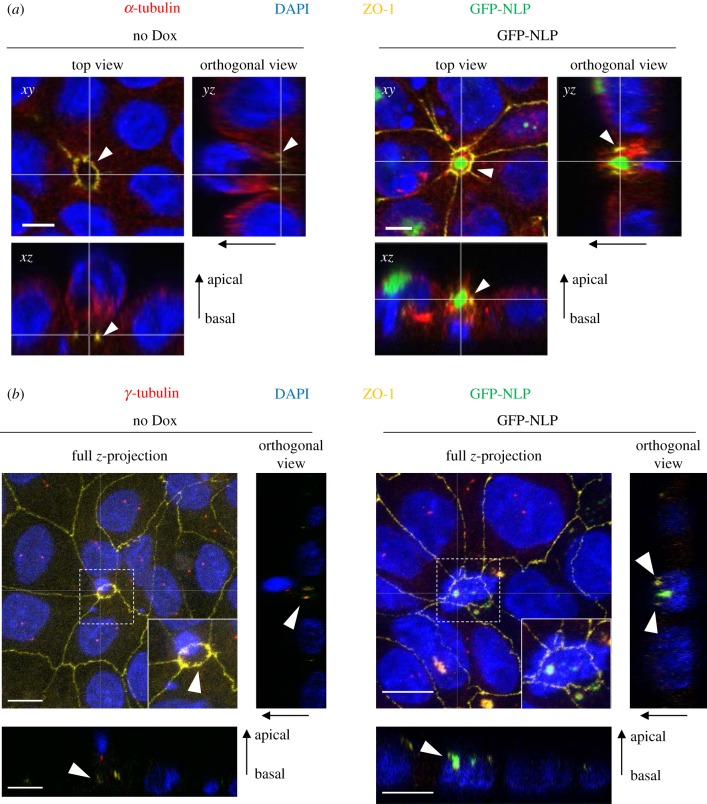
Positioning of MTs and centrosomes during basal cell extrusion. (*a*) Representative images show extrusion of damaged cells from 2D MDCK monolayer cultures that were induced (GFP-NLP) or not (no Dox) to express GFP-NLP for 24 h and the treated with etoposide. Cells were fixed, permeabilized, stained as indicated and examined by confocal fluorescence microscopy. GFP-NLP is shown in green and DNA in blue (DAPI staining). α-tubulin is pseudo-coloured in red and ZO-1 in yellow. Main panels show top views (*xy*). Corresponding orthogonal sections derived from 3D reconstructions of *z*-stacks are shown below (*xz*) and to the right (*yz*). The thin white lines illustrate the positions of the optical sections; epithelial polarity is indicated by arrows (apical/basal). The thin white lines illustrate the positions of the optical sections. White arrowheads mark the positions of contraction rings, as visualized by ZO-1 staining. Note that ZO-1 rings constrict either below (no Dox) or above (GFP-NLP) the nucleus of the extruding cell (visualized by DAPI staining), reflecting apical and basal extrusion, respectively. Scale bars = 5 µm. (*b*) The same experiment as in (*a*), except that antibodies against γ-tubulin were used to visualize centrosomes and full *z*-projections are shown. Dashed squares indicate the regions chosen for enlargements (insets). Scale bars = 10 µm.

The direction of cell extrusion depends upon the positioning of contractile actomyosin rings (figure 1c; electronic supplementary material, figure S2), which in turn depends on the assembly of MTs [35]. Of particular interest, it has previously been shown that deregulation of MT localization through mutation of the tumour suppressor protein APC can cause a change in the direction of cell extrusion from apical to basal [34]. This led us to further explore the relationship between NLP-induced centrosome aberrations and the disposition of MT arrays. Structural centrosome aberrations cause a profound reorganization of the MT cytoskeleton [21], and this was confirmed here by careful analysis of MT staining in relation to the positioning of ZO-1 ring contraction during cell extrusion (figure 5*a*; electronic supplementary material, figure S1b). Cells undergoing apical extrusion in control MDCK monolayers typically showed an accumulation of MTs underneath the nucleus, attesting to the ability of the MT cytoskeleton to undergo rearrangements (figure 5*a*, left panels). In stark contrast, cells undergoing basal extrusion in response to excess NLP showed a striking concentration of MTs around the structurally aberrant centrosomes, so that MTs were localized to a region between the aberrant centrosomes and the closing ZO-1 ring, with the nucleus positioned underneath (figure 5*a*, right panels). Likewise, the centrosome marker γ-tubulin was observed below the nucleus and well above the closing ZO-ring in cells extruding apically from wild-type epithelia (figure 5*b*, left panels), but trapped by the closing ZO-1 ring below the apical membrane in cells extruding basally in response to excess NLP (figure 5*b*, right panels). Considering that excess NLP anchors MTs within the apical region of polarized epithelial cells [21], the most straightforward interpretation of these observations is that NLP-induced structural centrosome aberrations restrict the repositioning of any cellular constituents that depend on MTs for their localization. This restriction probably affects S1P, the ligand in the SIP-S1PR2 signalling module, whose trafficking was previously shown to be regulated by MTs [28,30].

### 2.5. Overexpression of CEP131 induces spontaneous basal extrusion

**Figure 6.**
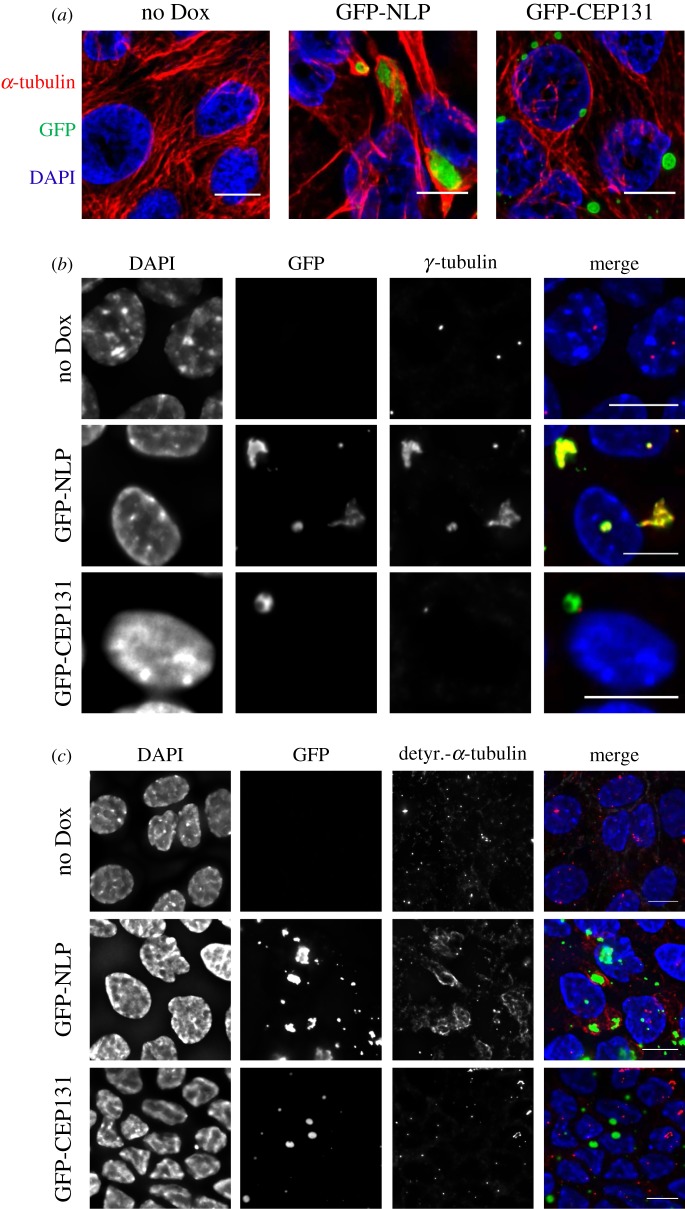
Structural centrosome aberrations caused by excess NLP or excess CEP131 are distinct. (*a*) Representative immunofluorescence images of MDCK cells cultured in 2D monolayers and stained for α-tubulin (red) and DNA (blue). Cells were not induced (no Dox) or induced to express GFP-NLP (green) or GFP-CEP131 (green) for 48 h. Scale bars = 10 µm. (*b*) MDCK cells were treated as in (*a*) and stained for γ-tubulin (red). Note that both GFP-NLP and GPF-CEP131 assemble at centrosomes (green), but only excess GFP-NLP also causes the accumulation of γ-tubulin. DNA was stained with DAPI (blue). Scale bars = 10 µm. (*c*) MDCK cells were treated as in (*a*) and stained for detyrosinated α-tubulin (red). Note that excess GFP-NLP (green) causes MT stabilization, as revealed by increased staining for detyrosinated α-tubulin, but excess GFP-CEP131 does not. DNA was stained with DAPI (blue). Scale bars = 10 µm.

**Figure 7.**
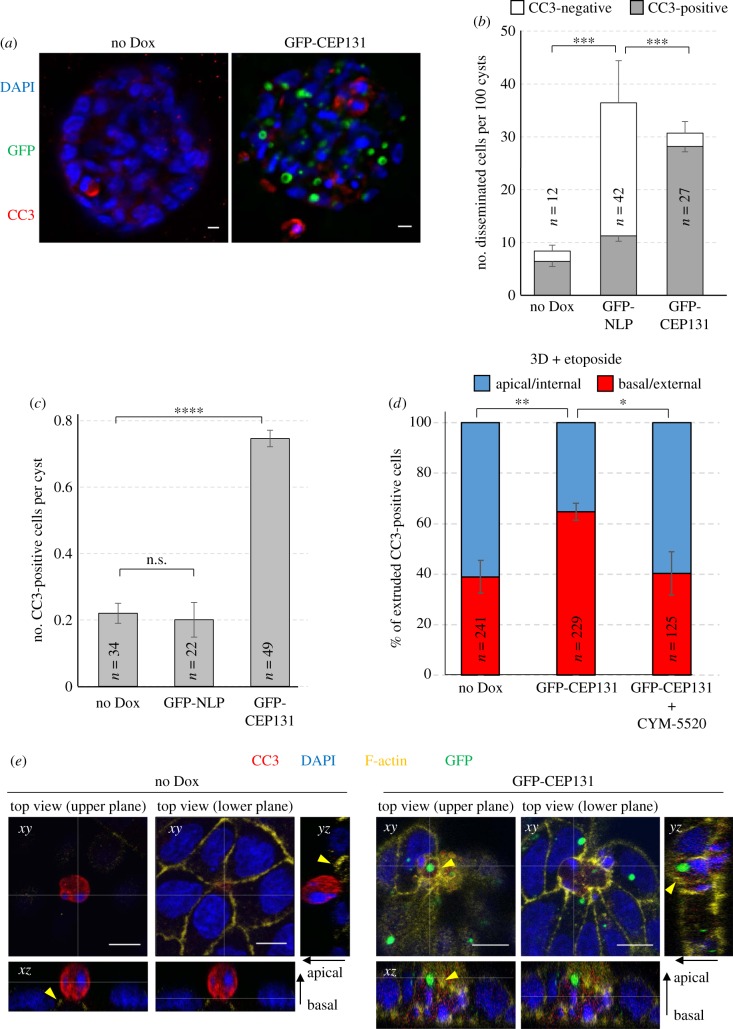
CEP131 triggers spontaneous basal cell extrusion. (*a*) Representative images show MDCK-derived cysts expressing GFP-CEP131 (right-hand panel) or not (left-hand panel). Cysts were stained for CC3 (red). GFP-CEP131 is shown in green and DNA in blue (DAPI staining). Scale bars = 10 µm. (*b*) Histogram shows the mean number of cells disseminated spontaneously (without etoposide treatment) from MDCK cysts expressing no transgene product (no Dox), GFP-NLP or GFP-CEP131. We note that the ‘no Dox' and ‘GFP-NLP' data were already shown in figure 1*b* (obtained from the same series of experiments as the ‘CEP131 data'). For each experiment, disseminated cells were classified according to CC3 staining and data are calibrated per 100 cysts. Grey bars: CC3-positive cells (apoptotic); white bars: CC3-negative cells. *n* refers to the numbers of disseminated cells detected in 155 control cysts (no Dox), 111 cysts expressing GFP-NLP and 66 cysts expressing GFP-CEP131^+^ (compiled from three independent experiments); error bars indicate +s.d. of the mean for CC3-negative cells and –s.d. of the mean for CC3-positive cells. *p*-values were derived from unpaired, two-tailed Student's *t*-test performed on CC3-positive cells. (*) indicates *p* < 0.05 and (****) indicates *p* < 0.0001. (*c*) Histogram shows the mean number of CC3-positive per MDCK cyst expressing no transgene product (No Dox), GFP-NLP or GFP-CEP131. We note that the ‘no Dox' and ‘GFP-NLP' data were already shown in figure 1*b* (obtained from the same series of experiments as the ‘CEP131 data'). *n* refers to the numbers of CC3-positive cells detected in the same cysts as described in (*b*) (compiled from three independent experiments); error bars indicate ±s.d. of the mean. n.s. indicates not significant and (****) indicates a *p* < 0.0001, as derived from unpaired, two-tailed Student's *t*-test. (*d*) Histogram indicates the mean percentages of CC3-positive cells extruded basally (red) versus apically (blue) from MDCK cysts that had been induced (GFP-CEP131) or not (No DOX) to express GFP-CEP131 for 48 h and then treated for 16 h with etoposide to trigger cell extrusion Where indicated, cysts were additionally treated with CYM-5520, an agonist of S1PR2. Bars represent means ± s.d. and *n* refers to the numbers of CC3-positive cells analysed (compiled from three independent experiments). *p*-values were derived from unpaired, two-tailed Student's *t*-test. (*) and (**) indicate *p* < 0.05 and less than 0.005, respectively. (*e*) Representative images show extrusion of damaged cells from 2D MDCK monolayer cultures that were induced (GFP-CEP131, right-hand panels) or not (no Dox, left-hand panels) to express GFP-CEP131 and treated with etoposide. Cells were fixed, permeabilized, stained for CC3 (red) and F-actin (yellow), and examined by confocal fluorescence microscopy. GFP-CEP131 is shown in green and DNA in blue (DAPI staining). Main panels show top views of the epithelium (xy sections) recorded at two different focal planes (upper/lower planes). Corresponding orthogonal sections derived from 3D reconstructions of *z*-stacks are shown below (*xz*) and to the right (*yz*). The thin white lines illustrate the positions of the optical sections; epithelial polarity is indicated on the right (apical/basal). Yellow arrowheads point to closing actomyosin rings. Scale bars = 10 μm.

The above results indicate that the overexpression of NLP not only causes structural centrosome aberrations but also favours basal extrusion of damaged cells. Whereas cell death occurs rarely in undisturbed cultures expressing excess NLP, it becomes a prominent feature upon induction of cell damage by etoposide. To determine whether basal extrusion might represent a more general response to structural centrosome aberrations, we tested the ability of a number of centrosome proteins to cause both structural centrosome aberrations and basal cell extrusion. Specifically, we examined the consequences of overexpressing CEP76, Ninein, γ-tubulin, FOP1, CEP120, CEP135 and CEP131 [26,44–47]. The results of this small screen led us to focus on CEP131 (also known as AZI1), a centrosome component implicated in ciliogenesis [47–49], cellular stress responses [50] and genome stability [51]. Moreover, CEP131 is frequently overexpressed in cancer [36,37]. We found that overexpression of GFP-CEP131 in MDCK monolayers caused striking structural centrosome aberrations in MDCK monolayers (figure 6*a*) as well as 3D MDCK cysts (figure 7*a*). Importantly, these CEP131-induced centrosome aberrations could readily be distinguished from NLP-induced aberrations, with regard to both their appearances and their properties (figures 6 and 7). Whereas both NLP and CEP131 accumulated at centrosomes, the enlarged structures formed in response to excess NLP were irregular in shape, but those formed by excess CEP131 appeared spherical, droplet-like (figure 6*a*). Moreover, whereas the NLP-induced structures accumulated γ-tubulin, consistent with earlier data [21,26], the CEP131-induced ones did not (figure 6*b*). Finally, only excess NLP caused the accumulation of detyrosinated α-tubulin, but excess CEP131 did not (figure 6*c*). As shown previously, the presence of detyrosinated α-tubulin correlates with enhanced MT stability [52,53] and increased cellular stiffness [23,54], and we emphasize that these properties are essential for the recently described budding of mitotic cells from epithelia [23].

**Figure 8.**
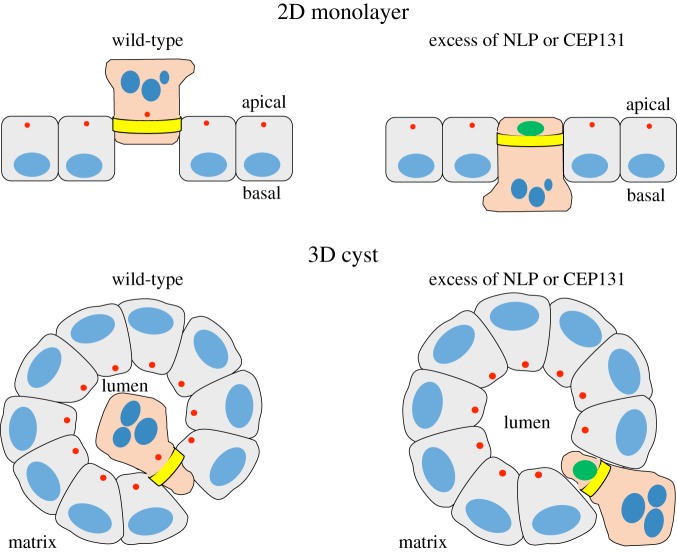
Schematic illustrating apical and basal extrusion events in 2D and 3D cultures. Cartoons depict apical and basal extrusion events from 2D monolayers (upper panel) and 3D cysts (lower panel), respectively. Nuclei are shown in blue, centrosomes in red, constricting actomyosin rings in yellow and the aberrant centrosomes induced by excess of NLP or CEP131 in green. In extruding cells, the nuclei are shown as apoptotic. The partial reversal in directionality of extrusion observed in response to NLP- or CEP131-induced centrosome aberrations is reminiscent of the phenotype caused by oncogenic mutations in K-Ras or APC.

Most importantly, NLP- and CEP131-induced centrosome aberrations caused strikingly distinct consequences. While overexpression of both proteins triggered frequent cell dissemination from MDCK cysts (figure 7*a,b*), most of the cells ‘budding' in response to NLP were alive (figure 7*b*) [23]. In stark contrast, the vast majority of the cells extruded from cysts in response to CEP131 were positive for CC3, indicating that they were undergoing apoptosis (figure 7*a,b*). In fact, while spontaneously dying cells were rarely observed in control MDCK cysts or in cysts harbouring structural centrosome aberrations induced by NLP, CC3-positive cells were commonly observed in response to overexpression of CEP131 (figure 7*c*). To determine whether the increased dissemination of dying cells caused by CEP131 overexpression only reflected an overall increase in apoptosis or, alternatively, an additional effect on the directionality of cell extrusion, we monitored the proportion of apoptotic cells that were extruded apically (into the lumen) or basally (into the surrounding matrix) after exposing MDCK cysts to etoposide. As shown above for NLP-induced centrosome aberrations (figure 4), CEP131-induced aberrations also caused a significant bias in favour of basal cell extrusion and, again, this phenotype could be reverted by the S1PR2-agonist CYM-5520 (figure 7*d*). Moreover, analysis of fixed MDCK monolayer cultures (figure 7*e*) as well as time-lapse experiments (figure 2*c*; electronic supplementary material, movie S4) confirmed that overexpression of CEP131 affects the positioning of actomyosin ring closure and the directionality of cell extrusion in a very similar way as overexpression of NLP (figure 8). Taken together, these results suggest that CEP131 overexpression induces structural centrosome aberrations that then trigger the basal extrusion of dying cells, even in the absence of any damaging agent.

## 3. Discussion

Centrosome aberrations occur in the vast majority of human tumours [55–57], and experimentally induced centrosome aberrations can trigger tumorigenesis in animals [12–15,27]. Taken together, this evidence strongly suggests that centrosome aberrations contribute to tumour development and cancer progression in human patients. At present, numerical centrosome aberrations are recognized to constitute a major cause of chromosome mis-segregation, implying that centrosome anomalies are likely to contribute to the aneuploidy and chromosomal instability of human cancers [7,10,11]. Additionally, centrosome aberrations cause disruption of tissue architecture, with potential implications for metastasis [20–23,58]. Of particular interest, recent studies have identified two different mechanisms through which centrosome aberrations may confer potentially invasive properties to cancer cells. These are, first, invadopodia formation [20,23] and, second, non-cell-autonomous dissemination (budding) of mitotic cells [23]. Here, we describe a third mechanism that establishes a potential link between centrosome aberrations and cell dissemination. Specifically, we document that structural centrosome aberrations, induced by overexpression of either NLP or CEP131, cause a reversal in the directionality of cell extrusion from epithelia, from apical to basal. This suggests that, similar to oncogenic mutations, centrosome aberrations may cause dissemination of potentially metastatic cells through basal cell extrusion.

The disposal of damaged cells into the lumen of a gland via apical extrusion depends on the cooperation between the cell to be extruded and the surrounding cells [59]. Whereas the surrounding cells are required to trigger the extrusion via the formation of characteristic rosette-like cellular arrangements, the directionality of extrusion is determined by mechanisms operating within the cell to be extruded [28,35]. Specifically, apical extrusion requires the active repositioning of contractile actomyosin rings in close proximity to the basement membrane and, inside the cell to be extruded, this repositioning depends on MT-dependent re-localization of S1P towards the basal compartment [30,33,34]. S1P then guides the placing of the contractile rings in neighbouring cells through interactions with S1PR2. In normal epithelia, this mechanism allows for apical elimination of damaged cells. However, in response to mutant K-Ras or APC, these pathways are blocked, resulting in a reversal of extrusion directionality [33,34]. A detailed understanding of the molecular mechanism(s) underlying basal cell extrusion induced by structural centrosome aberrations will require additional study, but our present results demonstrate that both NLP- and CEP131-induced centrosome aberrations block the re-localizations of both the MT and actomyosin cytoskeleton. Moreover, the basal cell extrusion triggered by overexpression of either NLP or CEP131 was sensitive to CYM5520, a potent and selective agonist of SP1R2, attesting to an involvement of SP1-SP1R2 signalling. This strongly suggests that structural centrosome aberrations trigger basal cell extrusion through mechanisms that are similar to, or at least overlap with, those used by mutant K-Ras [31,33] and APC proteins [34]. Moreover, considering that basal cell extrusion may share pathways involved in epithelial–mesenchymal transition (EMT) [29], it may be rewarding to further explore the relationship between centrosome aberrations and EMT.

The involvement of S1P-S1PR2 signalling in NLP- and CEP131-induced basal cell extrusion also contributes to distinguish this process from the recently described non-cell-autonomous dissemination of mitotic cells that is triggered by excess NLP [23]. In fact, while the S1PR2-agonist CYM-5520 interferes with basal extrusion of damaged cells in response to structural centrosome aberrations (figures 4 and 7*d*), this compound exerts no influence on the budding of mitotic cells that is triggered by excess NLP in the absence of etoposide [23]. Conversely, the Arp2/3 inhibitor CK-666 suppresses the live-cell dissemination that is triggered by NLP-induced centrosome aberrations [23], but does not influence the directionality of extrusion of damaged cells (figure 4). Taken together, these observations confirm that basal cell extrusion and mitotic cell budding involve distinct mechanisms [23].

In wild-type epithelia, etoposide causes the extrusion of damaged cells preferentially to the apical side, but, as shown here, the presence of NLP-induced structural centrosome aberrations introduces a significant bias in favour of basal cell extrusion. Attesting to the specificity of this phenotype, no such reversal of extrusion directionality was seen in response to PLK4-induced numerical centrosome aberrations or overexpression of the control protein CEP68. Remarkably, overexpression of CEP131 resulted in preferential basal cell extrusion even in the absence of etoposide treatment. In this case, virtually all extruded cells stained positive for CC3, indicating that CEP131-induced structural centrosome aberrations are prone to cause cell damage (figure 6*c*). This establishes a clear difference to NLP, whose overexpression does not detectably increase the frequency of cell death and instead results in dissemination of living cells (electronic supplementary material, figure S1a; figure 7*c*; see also [23]). We emphasize that the basal cell extrusion triggered by overexpression of CEP131, or by excess NLP in combination with etoposide, concerns damaged cells. Therefore, to play a role in metastasis, such cells would need to acquire additional mutations to counteract cell death and confer survival.

Both NLP and CEP131 are frequently overexpressed in human cancers. As shown previously, the structural centrosome aberration induced by overexpression of NLP in culture models [21,26,27] closely resembles those described in human breast cancer [55–57]. Moreover, the levels of NLP overexpression achieved in culture are comparable with those seen in human tumours [21,26,27,60,61]. Similarly, CEP131 is overexpressed in different types of human cancers, including hepatocellular cancer [37] and breast cancer [36]. In the latter case, the accumulation of CEP131 has been attributed to an excess of the CEP131-associated deubiquitinase USP9X [36]. As we show here, overexpression of both NLP and CEP131 results in strikingly enlarged centrosome-associated structures. Yet these structures display fundamentally distinct properties. First, whereas NLP-induced aberrations show irregular contours, CEP131-induced aberrations appear droplet-like, suggestive of an underlying phase transition [62–65]. In future, it will be interesting to compare the appearances of NLP- and CEP131-induced centrosome aberrations by electron microscopy and other structural approaches. Second, NLP-induced structural centrosome aberrations recruit γ-tubulin, indicating that the observed accumulation of MTs at enlarged centrosomes can be attributed to anchoring of MT minus ends [23]. By contrast, CEP131-induced aberrations fail to accumulate γ-tubulin. Finally, only excess NLP results in the accumulation of detyrosinated α-tubulin, indicative of MT stabilization and increased cellular stiffness [52–54], while no such phenotype is seen in response to excess CEP131. This is an important distinction, because the NLP-induced non-cell-autonomous dissemination of mitotic cells (budding) requires MT stabilization and increased cellular stiffness [23]. This readily explains why overexpression of CEP131 fails to cause budding.

In conclusion, our present study reveals an additional mechanism, basal cell extrusion, by which centrosome aberrations may cause dissemination of potentially metastatic cells. While basal cell extrusion has previously been linked to oncogenic mutations, we are not aware of previous studies implicating centrosome aberrations in the directionality of cell extrusion. Together with earlier studies [21,23], our present findings illustrate that seemingly similar structural centrosome aberrations can display fundamentally distinct properties, depending on the nature and composition of the centrosomal protein(s) that give rise to the anomaly. Specifically, the evidence suggests that different types of centrosome aberrations may contribute to metastatic cell dissemination through at least three fundamentally distinct mechanisms, invadopodia formation [20,23], non-cell-autonomous dissemination of mitotic cells [23] and basal cell extrusion (this study). This emphasizes the importance of establishing the precise molecular and structural properties of centrosome aberrations that prevail in different human tumours. Moreover, it raises the prospect that future molecular classification of centrosome aberrations may prove valuable in a clinical context. In particular, it is tempting to speculate that specific centrosome aberrations may in future prove to be of prognostic value for predicting chromosomal instability and/or a tumour's potential for metastasis.

## 4. Material and methods

### 4.1. Cell culture

MDCK II cells were provided by Inke Naethke (University of Dundee, UK) and grown in Minimum Essential Eagle Medium supplemented with 10% fetal calf serum (GE Healthcare, Chicago, IL, USA) and 5% Penicillin Streptomycin (Life Technologies, Carlsbad, CA, USA). The Phoenix ampho retroviral packaging cells and HEK293T cells were provided by Stefan Zimmermann and Ralph Wäsch, respectively (University Medical Center Freiburg), and grown in Dulbecco's modified Eagle's (DMEM) medium supplemented with 10% fetal calf serum, 1 mM sodium pyruvate (Life Technologies) and 5% Penicillin Streptomycin (Life Technologies). All cells were grown in a 37°C incubator with 5% CO_2_. Cultures were routinely tested for mycoplasma contamination by PCR, using growth media from high-density cultures as templates. Cysts derived from MDCK II were generated by plating single cells onto beds of Matrigel and propagated as described previously [23]. Cell extrusion was stimulated by treatments of MDCK monolayers or cysts with 10 µM etoposide (Sigma-Aldrich). The duration of etoposide treatment was 6 h for cells cultured in 2D monolayers and 16 h for cysts grown in 3D Matrigel. Anoikis was induced by culturing cells on dishes coated with poly-HEMA (Sigma-Aldrich), essentially as described previously [66]. In brief, culture dishes were coated twice with 20 mg ml^−1^ poly-HEMA in 95% ethanol and dried overnight. Poly-HEMA-coated dishes were then washed twice with PBS, before 2.5 × 10^6^ cells per 10-cm dish were seeded in complete medium. Prior to seeding, cells had been induced (+Dox) or not (−Dox) for GFP-NLP expression for 48 h. Cells were harvested after 24 h, before proteins were extracted and analysed by western blotting.

### 4.2. Generation of expression constructs and cell lines

Plasmids coding for mCherry-α-tubulin and mCardinal-ZO1-C-14 were PCR-amplified from pmCherry_*α*_tubulin_IRES_puro2 (kindly provided by Daniel Gerlich) [67] and from mCardinal-ZO-1-C-14 (gift from Michael Davidson; Addgene plasmid # 56179), respectively, and then ligated into pMXs-IRES-Blasticidin (Cell Biolabs Inc.). Doxycycline-inducible MDCK II cells were generated using the same two-step transduction strategy as described previously [21] and enriched by antibiotic selection using hygromycin (Life Technologies) at 1600 µg ml^−1^ and puromycin (Sigma-Aldrich) at 2 µg ml^−1^. Inducible MDCK II cells stably expressing mCherry-α-tubulin or mCardinal-ZO-1 were generated by retroviral transduction and subsequent selection in 5 µg ml^−1^ blasticidine (Novus Biologicals). GFP-CEP131 was PCR-amplified from pEGFP-C2-CEP131 plasmid [68] and ligated into pRetroX-Tight-Puro using Not1 and Mlu1 cloning sites. Expression of transgenes coding for EGFP-tagged centrosomal proteins was induced by treatment of cells with 2.5 µg ml^−1^ of doxycycline (Sigma-Aldrich) for 24 or 48 h, as indicated.

### 4.3. Western blotting

Cells were washed twice with PBS before total proteins were extracted using the following buffer: 50 mM Tris–HCl (pH 7.4), 150 mM NaCl, 0.5% IGEPAL (Sigma-Aldrich), 1 mM DTT, 5% glycerol, 50 mM NaF, 1 mM PMSF, 25 mM β-glycerophosphate, 1 mM orthovanadate, complete mini protease inhibitor cocktail (Roche Diagnostics, Basel, Switzerland). Proteins were separated by SDS–PAGE and transferred onto nitrocellulose membranes. Primary antibodies were directed against α-tubulin (clone DM1A, Sigma-Aldrich), NLP [26] or cleaved-caspase 3, CC3, (9661, Cell Signaling, Danvers, MA, USA); secondary antibodies were HRP-conjugated anti-mouse immunoglobulin (170-6516, Bio-Rad, Hercules, CA, USA) or anti-rabbit immunoglobulin (170-6515, Bio-Rad).

### 4.4. Fluorescence microscopy

For immunostaining of 2D cultures, MDCK II cells were grown on Ibidi 8 well microscopy slides, fixed for 15 min at room temperature using 3% paraformaldehyde in PBS, permeabilized 5 min using 0.5% Triton X-100 in PBS and blocked for 30 min in 2% BSA in PBS. For staining of γ-tubulin and detyrosinated-α-tubulin, cells were instead fixed and permeabilized by incubating them in 100% ice-cold methanol for 5 min at −20°C. Primary antibodies were directed against α-tubulin (T9026, Sigma-Aldrich), CC3 (9661, Cell Signaling), ZO-1 (D6L1E, Cell Signaling), γ-tubulin (T6557, Sigma-Aldrich) or detyrosinated-α-tubulin (ab48389, Abcam, Cambridge, UK). Secondary antibodies (all from Life Technologies) were AlexaFluor647 goat anti-mouse (A21236) and AlexaFluor568 donkey anti-rabbit (A10042). F-actin fibres were stained using AlexaFluor647-linked phalloidin at 33 nM (A22287, Life Technologies). For visualization of nuclei, DNA was stained using DAPI at 1 µg ml^−1^. Procedures for staining of 3D cysts were adapted from previously published protocols [69], as described [21,23].

Confocal images were acquired using a Leica SP5-II-MATRIX point scanning confocal microscope equipped with a 20×/0.70 HCX Plan Apo CS air objective and a 63×/1.40-1.60 HCX Plan Apo lambda blue oil immersion objective; 405 nm diode laser light used for detection of DAPI staining, 488 nm Argon laser light for visualization of GFP, 561 nm diode-pumped solid-state laser light for AlexaFluor568 stainings and 633 nm HeNe laser light AlexaFluor647 stainings. Image analyses, 3D reconstructions and final adjustments of confocal z-stacks (spacing 0.3 µm between confocal planes) were carried out using ImageJ or Imaris 8.1.2.

For time-lapse microscopy performed on 2D monolayers, cells were grown on collagen IV-coated Ibidi 8-well slides (80822, Ibidi, Germany) and expression of GFP-NLP or GFP-CEP131 was induced 48 h prior to the onset of recording; etoposide was added to the medium just before starting the time-lapse. For time-lapse experiments performed on 3D MDCK cysts, 5-day-old cysts were induced for expression of GFP-NLP and treated with 10 µM etoposide 6 h later. Then, the entire cysts were immediately monitored by time-lapse microscopy, with stacks of images spaced by 0.37 µm. Live-cell imaging analyses were carried out using a FEI MORE wide-field system (FEI Munich, Graefelfing, Germany) equipped with a 40×/0.95 U Plan S Apo air objective. For visualization of EGFP- or mCardinal signals, LEDs, combined with a quad bandpass filter, were used as light source to facilitate trans-illumination at 515/18 and 595/19 nm. Image acquisition was performed at 37°C, 5% CO_2_ and greater than 70% air humidity and acquisition cycles were repeated every 20 min or 18 min, as indicated. Image analyses were carried out using ImageJ, following deconvolution using Huygens Remote Manager 3.3.0-rc9.

For immunofluorescence experiments on cysts that had previously been recorded by time-lapse microscopy, samples were fixed at the end of the time-lapse experiments and stained as described above. To identify the relevant cysts, gridded bottom slides (80827 or 80826-G500, Ibidi) were used.

### 4.5. Classification of extrusion events

Confluent monolayers of MDCK II cells were induced to overexpress GFP-NLP, GFP-PLK4 or GFP-CEP68 by addition of doxycycline at 2.5 µg ml^−1^ for 24 or 48 h, as indicated. Extrusion of damaged cells from epithelial monolayers was then stimulated by addition of low-dose etoposide (10 µM) for 6 h, as described previously [34]. Extrusion events could readily be identified by the formation of typical rosette-like configurations of cells surrounding the individual damaged cells, and the characteristic enrichment of F-actin and ZO-1, as described previously [30,33]. Extrusion events were classified as basal or apical cell extrusions, depending on the positioning of the contractile ring relative to apoptotic DNA. In cases of basal extrusions, closure of the ring occurred in the apical part of the epithelial layer, resulting in the trapping of apoptotic DNA underneath the contractile ring. In cases of apical extrusion, closure of the ring occurred in the basal part of the epithelial layer. To determine the directionality of cell extrusion from 3D MDCK cysts, these were treated with doxycycline for 48 h (or not) to induce transgene expression and then challenged with 10 µM for 16 h to stimulate cell extrusion. Then, they were fixed and stained for CC3 to visualize apoptotic cells. CC3-positive cells within the surrounding matrix (but in very close proximity to the cyst) were classified as basal extrusions; apoptotic cells located within the cyst (but just underneath the external cell layer forming the cyst) were classified as apical extrusions. Confidence in this classification is supported by time-lapse experiments showing that dissemination of etoposide-induced apoptotic cells occurs through basal extrusions (as illustrated in figure 3). Inhibitors were used at the following concentrations: CK-666 (Sigma-Aldrich) at 50 µM and CYM-5520 (Sigma-Aldrich) at 10 µM. For rescue experiments, doxycycline and drugs were added simultaneously.

### 4.6. Statistical analyses

Sample sizes (*n*) were chosen to allow detection of statistically significant differences between subgroups within biological replicates. For statistical comparison of groups, two-tailed Student's *t*-tests were performed, assuming statistical significance for *p* < 0.05.

## Supplementary Material

Supplementary figures S1-S3

## Data accessibility

This article has no additional data.
