# Supplementary material for: Structural centrosome aberrations sensitize polarized epithelia to basal cell extrusion
Source: Open Biol. 2018 Jun 13;8(6):180044. doi: 10.1098/rsob.180044 (PMC6030118; doi:10.1098/rsob.180044)
Supplement: Supplementary figures S1-S3 [file rsob180044supp1.pdf]

OPEN BIOLOGY

Electronic Supplementary Material

Structural centrosome aberrations sensitize polarized epithelia to basal cell extrusion

(10.1098/rsob.20160044)

Olivier Ganier, Dominik Schnerch and Erich A. Nigg

Figure S1: Impact of induction of cell death on 2D MDCK cultures.

(a) Western blots were performed on total proteins extracted from MDCK cultures induced (+ Dox) or not (- Dox) to express GFP-NLP for 72 hours. Where indicated, anoikis was induced by seeding cells on PolyHema coated wells. Primary antibodies were anti-NLP (to illustrate level of expression of transgene product), anti-CC3 (to demonstrate apoptosis) and anti-α-tubulin (loading control).

(**b**) Representative images show extrusion of damaged cells from 2D MDCK monolayer cultures that had been induced (GFP-NLP, lower panels) or not (No Dox, upper panels) to express GFP-NLP for 48 hours and then treated with etoposide for 6 hours. Cells were fixed, permeabilized, stained for CC3 (red) and F-actin (yellow), and examined by confocal fluorescence microscopy. GFP-NLP is shown in green and DNA in blue (DAPI staining). Main panels show top views of the epithelium (xy sections) recorded at two different focal planes (upper/lower planes). Corresponding orthogonal sections derived from 3D reconstructions of z-stacks are shown below (xz) and to the right (yz). The thin white

lines illustrate the positions of the optical sections; epithelial polarity is indicated by arrows (Apical/Basal). Yellow arrowheads point to closing actomyosin rings. Scale bars = 10μm.

Figure S2: NLP-induced centrosome aberrations favor basal extrusion through interference with MT cytoskeleton.

The upper panel shows an overview (3D reconstruction) of an MDCK-derived epithelium induced to express GFP-NLP for 24 hours and then treated for 6 hours with etoposide to trigger cell extrusion. Cells were immunostained for ZO-1 (pseudo-colored in yellow) and α-tubulin (pseudo-colored in red) and examined by confocal fluorescence microscopy. GFP-NLP is shown in green and DNA in blue (DAPI staining). Lower panels show enlarged images of the boxed area. A top view (xy) is shown to the left, and corresponding orthogonal section derived from 3D reconstructions of z-stacks to the right (xz). The thin white lines illustrate the positions of the optical sections; epithelial polarity is by the arrow (Apical/Basal). White arrowheads point to closing actomyosin ring. Scale bar = 5μm.

Figure S3: Contraction of actin rings and closure of ZO-1 junctions around extruding cells

Representative images show extrusion of damaged cells from 2D MDCK monolayer cultures that had been induced (GFP-NLP, lower panels) or not (No Dox, upper panels) to express GFP-NLP and treated with etoposide. Cells were treated as described in the legend to Supplemental Figure 2**,** except that F-actin (pseudo-colored in red) was stained instead of α-tubulin. Main panels show top views (xy) and corresponding orthogonal sections (xz: Ortho. View) derived from 3D reconstructions of z-stacks are shown below. The thin white lines illustrate the positions of the optical sections; epithelial polarity is indicated on the right (Apical/Basal). White arrows point to nuclei of cells about to be extruded. The upper panels (No Dox) show a representative example of apical extrusion, while the lower panels (GFP-NLP) show a representative example of basal extrusion. We emphasize that each stage depicted here represents a different cell about to be extruded. For purely illustrative purposes,

these images were then arbitrarily arranged to suggest a sequence of events, labeled as ‘ring formation’, ring closure early’, ‘ring closure late’ and ‘junction sealing’. Scale bars = 5μm.

Figure S1

(a)

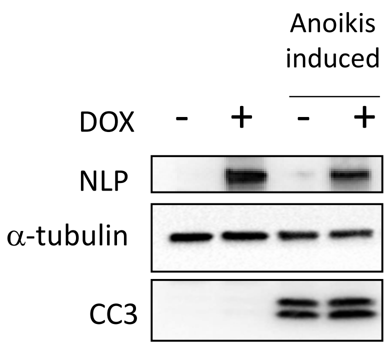
Anoikis induced
DOX - + - +
NLP
α-tubulin
CC3

(b)

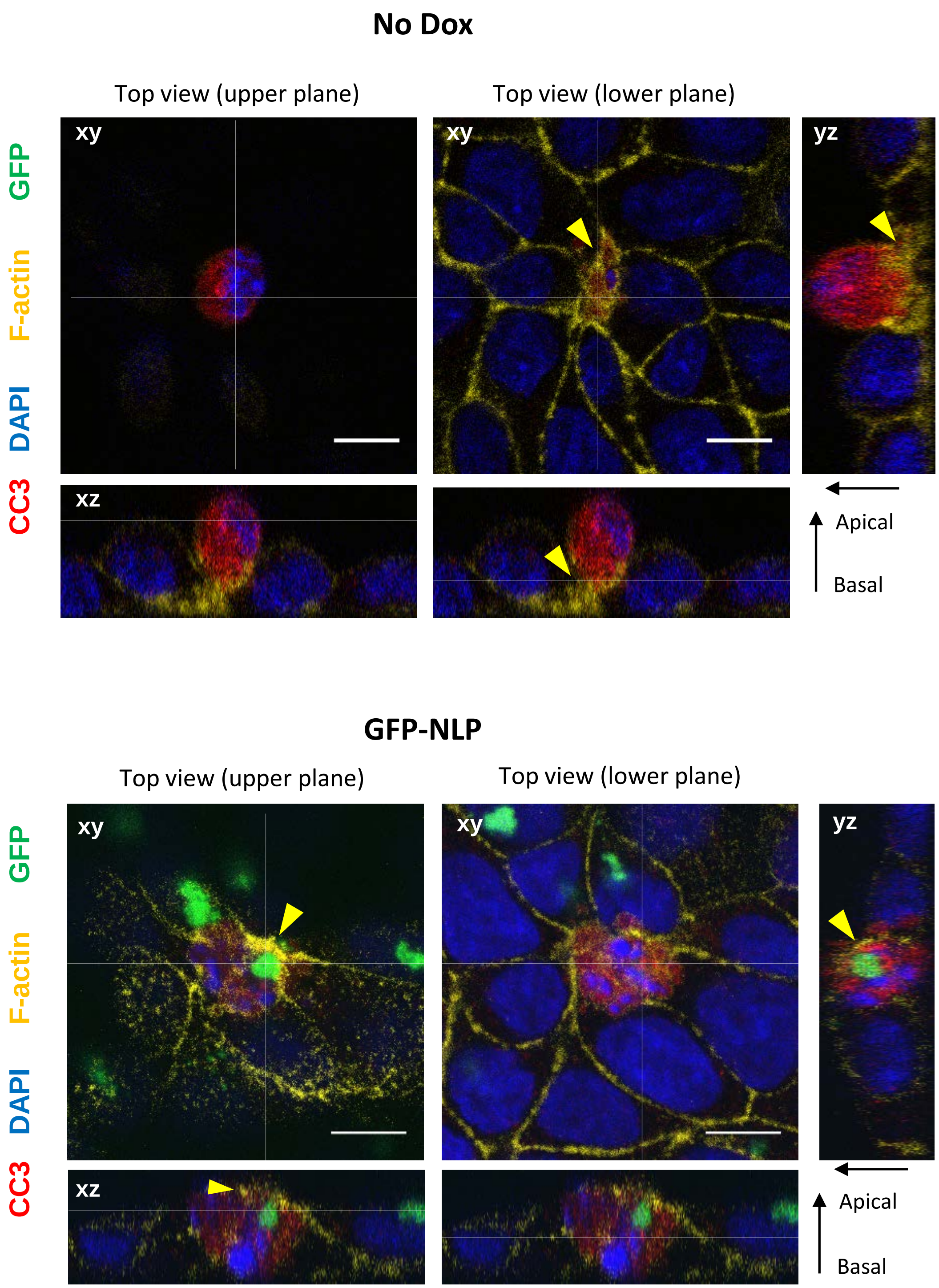
No Dox
Top view (upper plane)
Top view (lower plane)
xy
xy
yz
xz
GFP
F-actin
DAPI
CC3
Apical
Basal
GFP-NLP
Top view (upper plane)
Top view (lower plane)
xy
xy
yz
xz
GFP
F-actin
DAPI
CC3
Apical
Basal

Figure S2

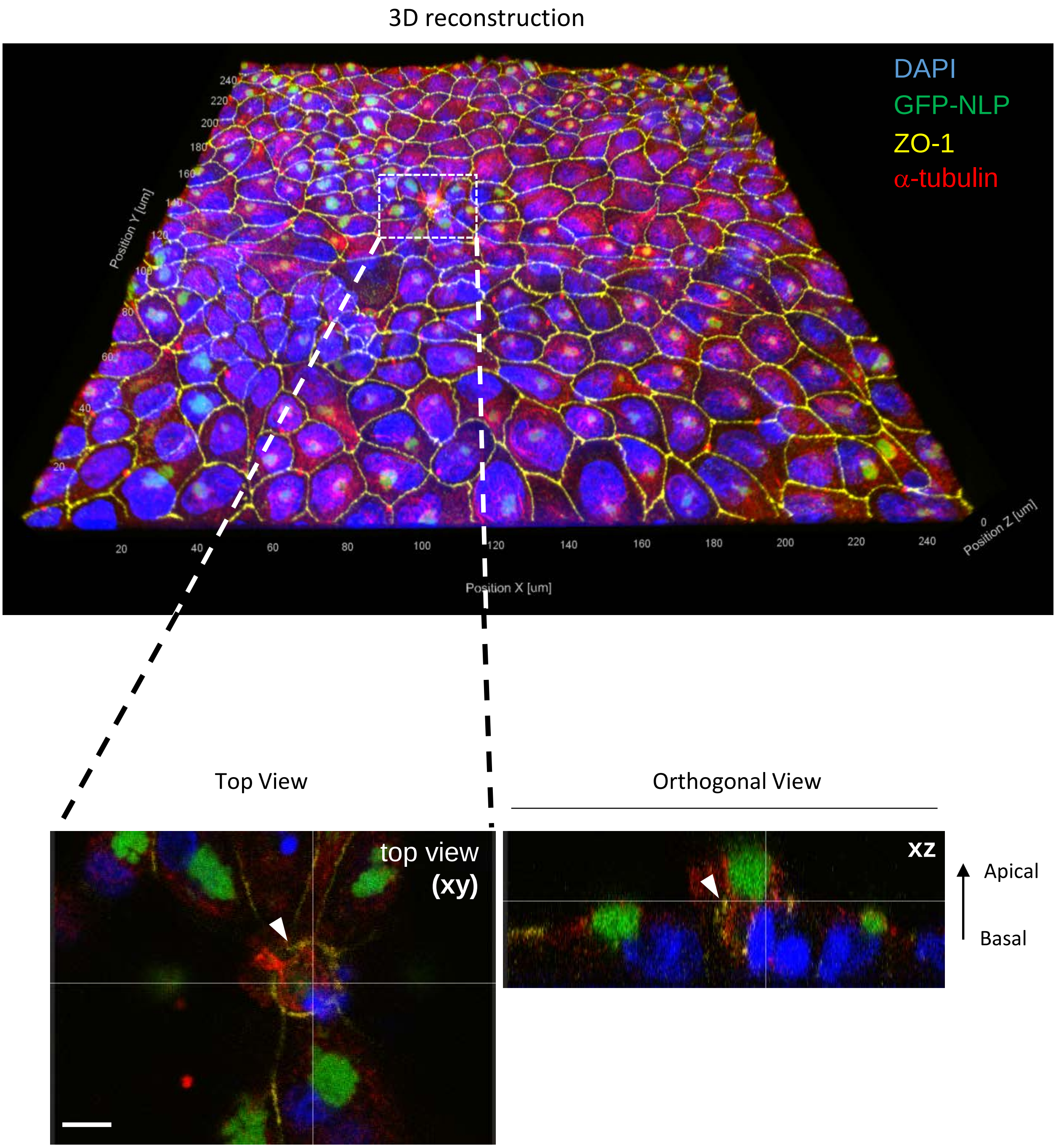

3D reconstruction
DAPI
GFP-NLP
ZO-1
α-tubulin
Position Y [um]
Position X [um]
Position Z [um]
Top View
Orthogonal View
top view
(xy)
xz
Apical
Basal

Figure S3

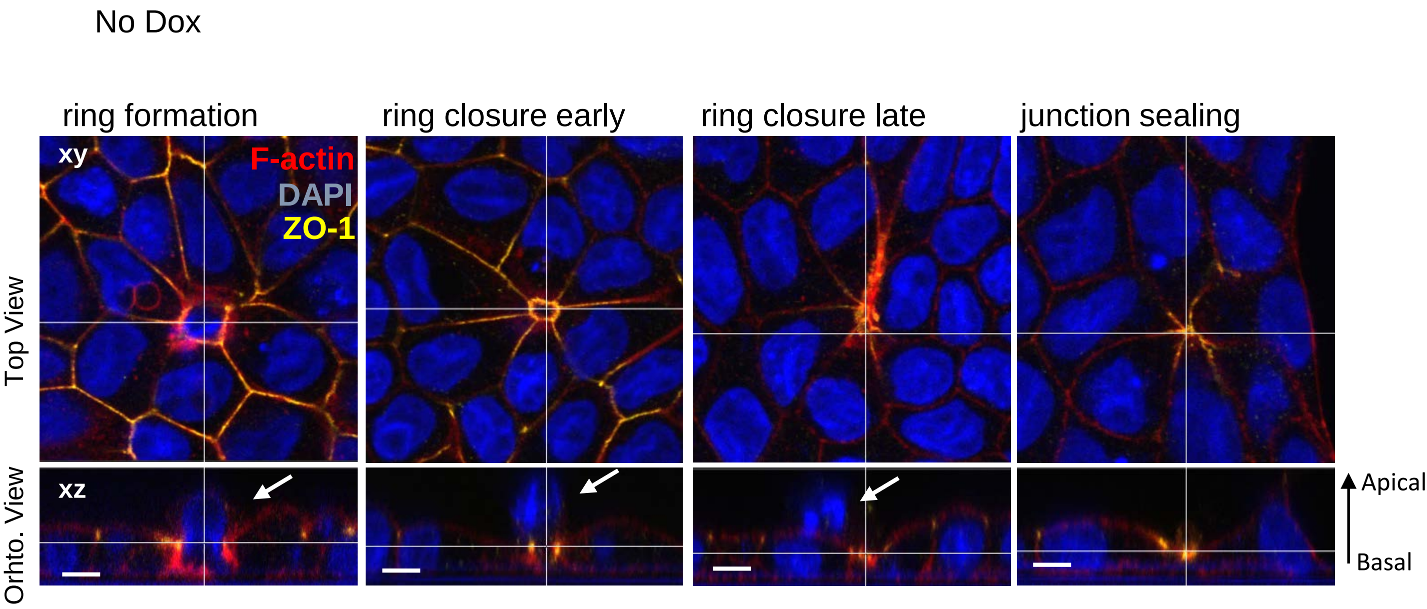
No Dox
ring formation
ring closure early
ring closure late
junction sealing
xy
F-actin
DAPI
ZO-1
Top View
xz
Orhto. View
Apical
Basal

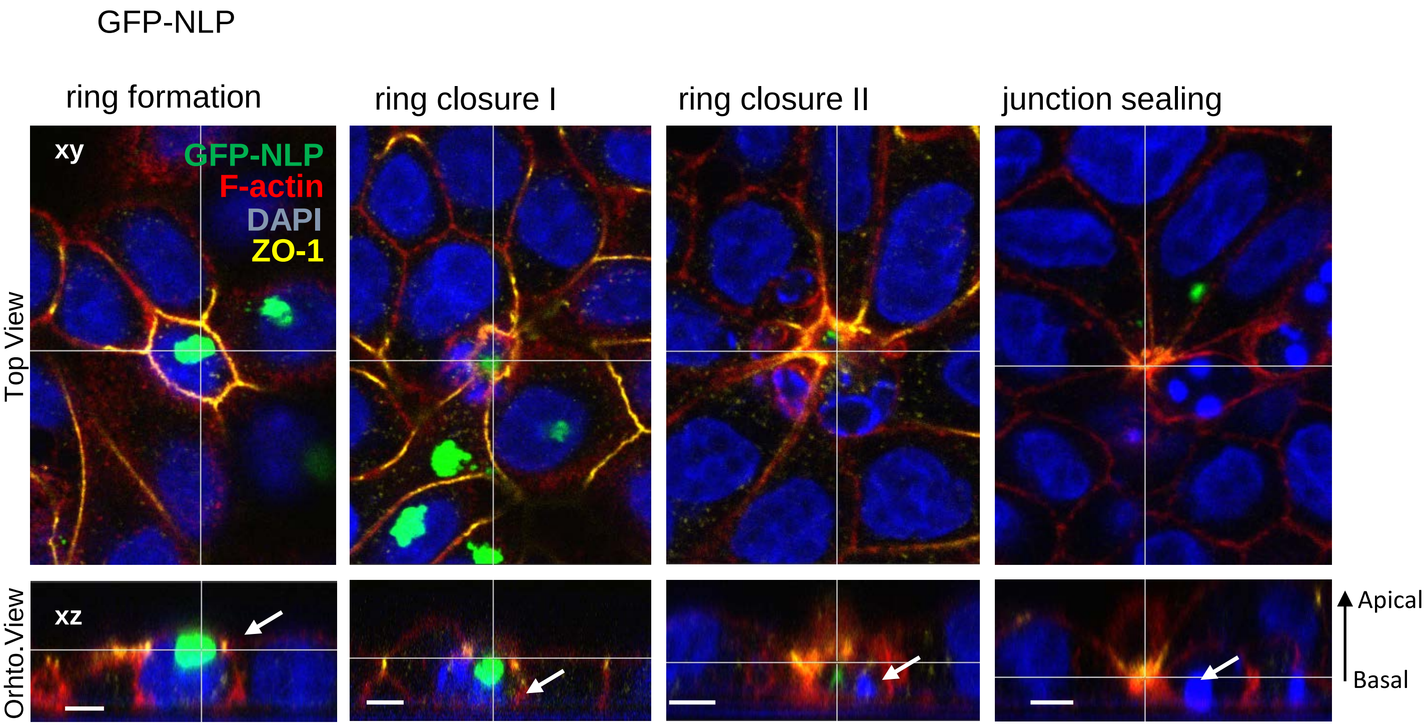
GFP-NLP
ring formation
ring closure I
ring closure II
junction sealing
xy
GFP-NLP
F-actin
DAPI
ZO-1
Top View
xz
Orhto.View
Apical
Basal
